# Utilization of Tea Polyphenols as Color Developers in Reversible Thermochromic Dyes for Thermosensitive Color Change and Enhanced Functionality of Polyester Fabrics

**DOI:** 10.3390/molecules29204944

**Published:** 2024-10-18

**Authors:** Weimian Zhou, Qun Yang, Sixuan Tao, Jin Cui, Jie Zhu, Siyu Zhou, Ruimiao Li, Juan Su, Ning Zhang, Lihui Xu, Hong Pan, Jiping Wang

**Affiliations:** 1 School of Textiles and Fashion, Shanghai University of Engineering Science, Shanghai 201620, China; zhouweimian2022@163.com (W.Z.);; 2 Key Laboratory of Textile Fiber and Products (Wuhan Textile University), Ministry of Education, Wuhan 430200, China; 3 Shanghai Engineering Research Center for Clean Production of Textile Chemistry, Shanghai 201620, China; 4 Shanghai Evershine Co., Ltd., Shanghai 201600, China

**Keywords:** reversible thermochromic dyes, tea polyphenols, polyester, phase change energy storage, smart textile

## Abstract

Thermochromic textiles possess the capability to indicate ambient temperature through color changes, enabling real-time temperature monitoring and providing temperature warnings for body heat management. In this study, three thermochromic dyes—blue, red, and yellow—were synthesized using crystalline violet lactone (CVL), 6′-(diethylamino)-1′,3′-dimethyl-fluoran (DDF), and 3′,6′-dimethoxyfluoran (DOF) as leuco dyes, respectively, with biomass tea polyphenol serving as the color developer and tetradecanol as the phase change material. The chemical structures of these dyes were characterized using UV spectroscopy, infrared spectroscopy, Raman spectroscopy and ^1^H NMR. The thermochromic mechanisms were investigated, revealing that the binding bonds between the leuco dyes and the color developer broke and reorganized with temperature changes, imparting reversible thermochromic property. Polyester fabrics were dyed using an impregnation method to produce three reversible thermochromic fabrics in blue, red, and yellow. The structure and properties of these fabrics were analyzed, showing a significant increase in the UPF value from 26.3 to approximately 100, indicating enhanced UV resistance. Water contact angle measurements revealed that the contact angle of undyed polyester fabrics was 139°, while that of dyed polyester fabrics decreased by about 40°, indicating improved hydrophilicity. Additionally, the fabric inductive static tester showed that the static voltage half-life of dyed polyester fabric was less than 1 s, demonstrating a significant antistatic effect. Infrared thermal imaging results indicated that during the warming and cooling process, the thermochromic polyester fabric exhibited specific energy storage and insulation effects at 38 °C, close to the human body temperature. This study presented a novel approach to developing smart color-changing textiles using biomass-derived thermochromic dyes, offering diverse materials for personal thermal management, and intelligent insulation applications.

## 1. Introduction

Color is a visual effect of light perceived through the eye, brain, and life experiences. Innovative color-changing materials have garnered significant attention due to their environmental adaptability, altering color in response to ambient temperature, humidity, pH, and other factors. The reversible color change or discoloration based on temperature variation is known as thermochromism. Thermochromic materials have been widely utilized in military applications, intelligent windows, architectural coatings, and textiles [1,2,3,4]. In extreme weather conditions, the thermal homeostasis of the human body can be disrupted. Thermochromic garments can function as temperature sensors, transmitting thermal signals to the wearer and monitoring body temperature in real-time [5,6]. Tarek M. Abou Elmaaty et al. [7] have printed thermochromic pigments (blue and red) on specially designed cotton children’s garments using flat screen-printing techniques. The garments exhibited noticeable color changes in response to body temperature, maintaining fastness to light, washing, and rubbing. Liu et al. [8] utilized biomass-based three-component thermochromic dyes for dyeing linen fabrics by vacuum impregnation. The dyed fabrics demonstrated changes in color, surface wettability, strength, and thermal insulation with the temperature change. Contrastingly, Yu et al. [9] developed conductive fibers with thermochromic effects through a combination of Joule heating and solar absorption modulation. These fibers, capable of electro-thermal conversion, can monitor the thermal environment of the human body. Silk, known for its excellent mechanical properties and breathability compared to synthetic polymer fibers, was used by Wang et al. [10] to create high-performance thermochromic silk fabrics. These fabrics, produced through a cost-effective, sustainable, and efficient finishing process, exhibited excellent thermochromic response properties.

Three-component thermochromic materials typically consist of a leuco dye, a color developer (electron donor), and a phase change solvent carrier. During dye synthesis, the lactone ring of leuco dye broke as the temperature increased, resulting in electron loss and complexation with the color developer to form a colored complex. At the same time, organic solvents served as equilibrium media for these three-component thermochromic materials. Among color developer systems, bisphenol A (BPA) has been widely used due to its structural advantages, ability to enhance the conversion rate of thermochromic dyes, and low cost [11,12]. However, BPA’s chemical structure is similar to estrogen, allowing it to bind to estrogen receptors (ER) and disrupt natural endocrine functions, leading to biotoxic effects such as breast cancer, endometriosis, and infertility [13,14]. Consequently, the production and use of BPA have been severely restricted to minimize adverse health effects. Several new BPA analogues have been developed and widely adopted as replacements. Tea polyphenols, as a biomass material with multiple phenolic hydroxyl groups, can be compounded with leuco dyes to achieve thermochromic effects, making them a promising alternative to BPA. Additionally, phase change materials (PCMs) can store and release latent heat during the phase transitions and were widely used in thermal management and storage [15]. Solid/liquid PCMs offered significant advantages, including high latent heat storage capacity and minimal volume change during phase transitions [16]. For example, tetradecanol (TD) is an essential organic solid/liquid PCM with a large storage capacity and a phase change temperature close to human body temperature (approximately 38 °C). Therefore, it has been extensively used in residential air conditioning, solar heating, waste heat recovery, and building energy efficiency [17,18,19,20].

Polyester fabrics exhibit excellent wrinkle resistance and shape retention properties. Additionally, polyester fibers possess high strength and elastic recovery, contributing to the fabric’s robustness and durability. However, they also present challenges, such as poor moisture absorption and susceptibility to static electricity. To address these issues, researchers have implemented several modifications. Zhang et al. [21] enhanced the hydrophilic and hygroscopic properties of polyester fabrics by combining pozzolanic materials with polydopamine-modified polyester. Furthermore, Fan et al. [22] utilized a novel graphene oxide-doped disperse dye for one-bath dyeing of polyester fabrics, imparting antistatic properties. The reduction of graphene oxide at 2% for 30 min achieved a class A antistatic standard, thereby improving the performance and suitability of polyester fabrics.

In this study, three thermochromic dyes—blue, red, and yellow—were synthesized using crystalline violet lactone (CVL), 6′-(diethylamino)-1′,3′-dimethyl-fluoran (DDF), and 3′,6′-dimethoxyfluoran (DOF) as leuco dyes, respectively. Biomass tea polyphenol served as the color rendering agent, while tetradecanol was utilized as the phase-change material. The chemical structures and thermochromic mechanisms of these dyes were thoroughly investigated. The synthesized thermochromic dyes were applied to polyester fabrics via the impregnation method. The thermal stability, hydrophilicity, hydrostatic half-life, and other properties of these three thermochromic polyester fabrics were evaluated. This environmentally friendly approach to creating thermochromic fabrics using biomass-derived dyes presented a novel pathway for intelligent color-changing textiles, offering innovative thermal insulation and enhancing diversity in personal heat management materials.

## 2. Results and Discussion

### 2.1. Structure of Biomass Thermochromic Dyes and Thermochromic Mechanism

**Figure 1:**
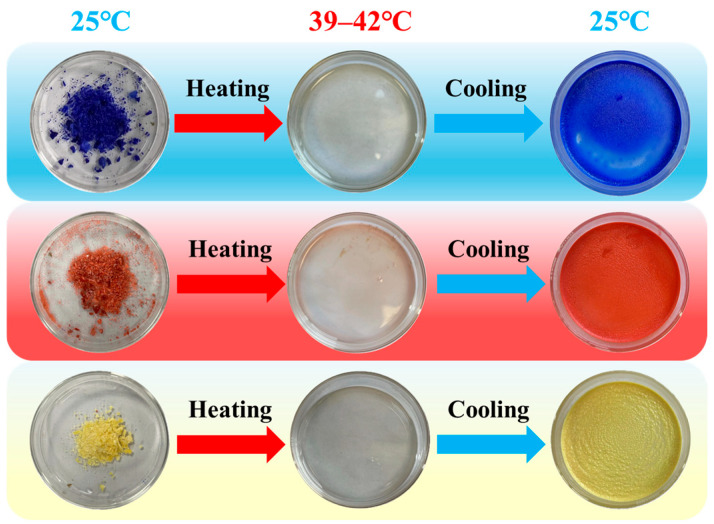
Temperature thresholds for color transitions in three reversible thermochromic dyes.

The reversible thermochromic dyes’ behavior was primarily influenced by the melting point of the phase change solvent. To determine the color change temperatures of the synthesized blue, red, and yellow dyes, a series of heating experiments were conducted with real-time monitoring. The results indicated that the discoloration temperatures for the three dyes fell within the range of 39–42 °C, which aligned with the phase transition temperature of the solvent tetradecanol, as illustrated in Figure 1. A reversible thermochromic system consists of leuco dyes, a color-developer and a cosolvent [23]. The interaction among these components is pivotal, with tetradecanol playing a crucial role in modulating the thermochromic response of the dyes.

**Figure 2:**
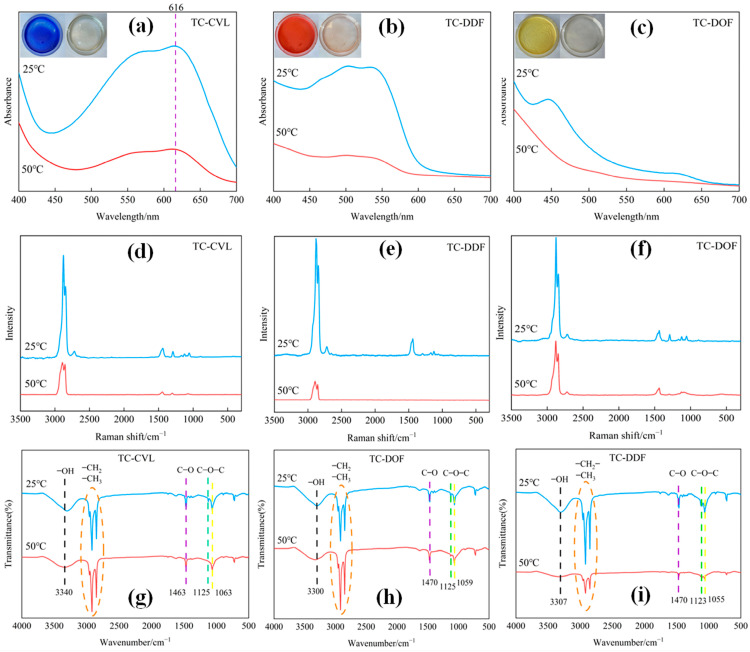
Structural analysis of three thermochromic dyes at 25 °C and 50 °C, respectively: (**a**–**c**) UV absorption spectra; (**d**–**f**) Raman spectra; (**g**–**i**) FTIR spectra.

The UV absorption spectra of the three biomass thermochromic dyes at 25 °C and 50 °C were analyzed using in situ UV spectroscopy. As shown in Figure 2a–c, the dyes TC-CVL (Figure 2a), TC-DDF (Figure 2b) and TC-DOF (Figure 2c) exhibited distinct absorptions at 560–630 nm, 490–550 nm, and 425–470 nm, respectively, indicating the color of blue, red, and yellow at 25 °C. Upon increasing the temperature to 50 °C, the absorbance at these wavelengths decreases significantly. The absorbance at 560–630 nm, 500–550 nm, and 440–470 nm became comparable to other wavelengths, suggesting that the chromophore groups of TC-CVL, TC-DDF, and TC-DOF were disrupted upon heating, rendering the dyes colorless. This observation aligned with the macroscopic appearance of the dyes, which are blue, red, and yellow at lower temperatures but become colorless upon heating (Figure 2a–c, upper left corner of the curve). 

The results shown in Figure 2d–f were in situ Raman spectroscopy at 25 °C and 50 °C, which indicated significant absorption near 2800 cm⁻^1^ at 25 °C, suggesting the presence of quinone and C=N structures. However, at 50 °C, the peak intensities near 2800 cm^−1^ decreased significantly, indicating temperature-induced changes in quinone and C=N structures. Additionally, changes in C=O vibration induced by alterations in the lactone ring structure at 1500 cm^−1^ were also observed in the Raman figure. The C–O–C vibration absorption peaks at 1060 cm^−1^ and 1120 cm^−1^ of the three dyes were close to the vibration absorption peaks in the infrared spectrum.

Figure 2g–i shows in situ infrared spectroscopy of the three dyes at 25 °C and 50 °C. For instance, in Figure 2d for TC-CVL, the broad peak at 3340 cm^−1^ was primarily attributed to the –OH group of the phase change solvent tetradecanol. This peak also indicates the presence of intermolecular and intramolecular hydrogen bonds within the thermochromic dye system. At 25 °C, the lactone group of CVL formed intramolecular hydrogen bonds with the phenolic hydroxyl group of tea polyphenol, resulting in the formation of the TC-CVL colored complex. As the temperature increased from 25 °C to 50 °C, the intensity of the broad peak at 3340 cm^−1^ diminished, indicating a weakening of intermolecular and intramolecular hydrogen bonding in the thermochromic dyes. This change was due to the transformation of the tetradecanol from a solid to a liquid state. In liquid tetradecanol, CVL and tea polyphenols dissolved and existed as two colorless complexes. The C–O–C vibrational absorption peaks of CVL at 1125 cm^−1^ and 1063 cm^−1^, along with the peaks at 1390 cm^−1^ and 1540 cm^−1^, correspond to the C–O asymmetric stretching vibrations associated with the ring-opening carboxylate structure in CVL [24]. These observed peaks remained largely unaffected by variations in temperature.

**Figure 3:**
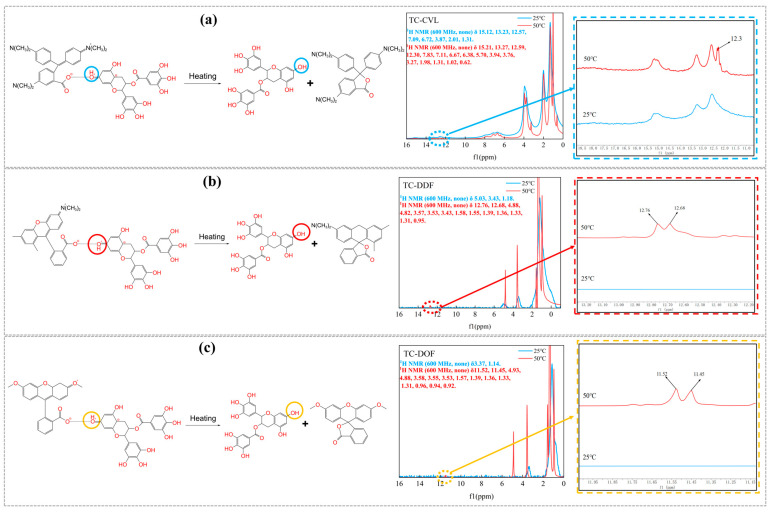
^1^H NMR spectra of three thermochromic dyes at 25 °C and 50 °C: (**a**) TC-CVL; (**b**) TC-DOF; (**c**) TC-DOF.

To further verify the structure of thermochromic dyes and the changes in their molecular structure before and after heating, the three dyes—TC-CVL, TC-DDF, and TC-DOF were characterized using a solid-state NMR at 25 °C and 50 °C, respectively, as shown in Figure 3. TC-CVL in Figure 3a as an example, the absorption peaks of each hydrogen atom at 25 °C were δ15.12 ppm, δ13.23 ppm, δ12.57 ppm, δ7.09 ppm, δ6.72 ppm, δ3.87 ppm, δ2.01 ppm, and δ1.31 ppm. In contrast, at an elevated temperature of 50 °C, the absorption peaks were δ15.21 ppm, δ13.27 ppm, δ12.59 ppm, δ12.30 ppm, δ7.83 ppm, δ7.11 ppm, δ6.67 ppm, δ6.38 ppm, δ5.70 ppm, δ3.94 ppm, δ3.76 ppm, δ3.27 ppm, δ1.98 ppm, δ1.31 ppm, δ1.02 ppm, and δ0.62 ppm. Based on the structural analysis of CVL and tea polyphenols, the peak at δ12.3 ppm corresponded to the phenolic hydroxyl active hydrogen of tea polyphenols. This was attributed to the phase transition of tetradecanol from solid to liquid state at 50 °C, which weakened the interaction between CVL and tea polyphenols. Consequently, the conjugated colorimetric structure was dissociated, and the hydrogen bonds between CVL and tea polyphenols broke as the temperature increased. The phenolic hydroxyl active hydrogen of tea polyphenols was influenced by the CVL conjugated structure, leading to a decrease in the extra-nuclear electron cloud density, a weakened shielding effect, an increased effective magnetic field, and a higher chemical shift. The analysis of Figure 3b,c indicated that TC-DDF and TC-DOF exhibited similar changes. This indicated that the three thermochromic dyes—TC-CVL, TC-DDF, and TC-DOF exhibited reversible thermochromic behavior. This behavior was facilitated by the disruption and subsequent reformation of hydrogen bonds between the cryptochromic dyes and tea polyphenols [25].

**Figure 4:**
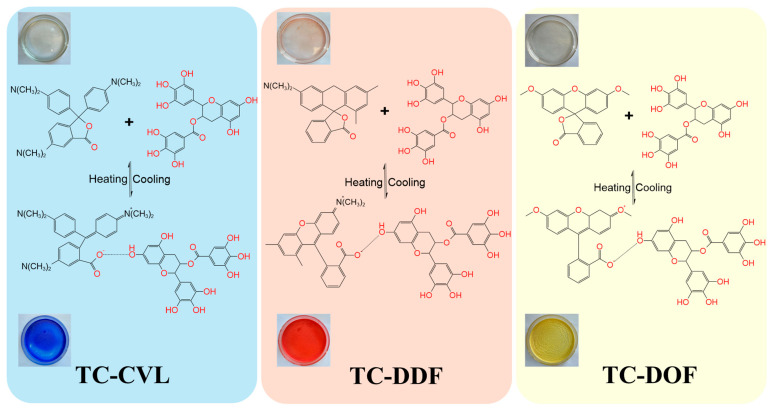
Molecular structure changes of three thermochromic dyes—TC-CVL, TC-DDF, and TC-DOF with heating and cooling.

The mechanism of thermochromic change can be described as follows: At low temperatures, the binding force between the leuco dyes and the color developer was strong. With the temperature increased or decreased, the hydroxyl groups (–OH) in tetradecanol underwent reversible transformations with quinone, while the lactone ring transformed with an open-loop acid by gaining or losing H^+^ ions. These transformations involved hydrogen transfer isomerization. The conjugation degree of the system was adjusted through associations and dissociations facilitated by hydrogen bonds between the carbonyl oxygen, hydroxyl oxygen, and hydroxyl hydrogen in open-loop acid, as well as hydroxyl oxygen and hydroxyl hydrogen in mixed alcohols (tetradecanol). Additional electrical effects between the carbonyl carbon in the open-loop acid and hydroxyl oxygen in tetradecanols were used and played a role in achieving a “crystallization-melting” cycle of eutectic phase within a specific heating-cooling temperature range [26]. The structural changes under varying temperatures are illustrated in Figure 4. For biomass thermochromic dyes, the lactone ring of the cryptochrome dye opened under the influence of temperature, forming a quinone-like structure. This structural transformation altered the coupling system of the dye, resulting in a color change.

### 2.2. Reversible Thermochromism of Dyed Polyester Fabric 

**Figure 5:**
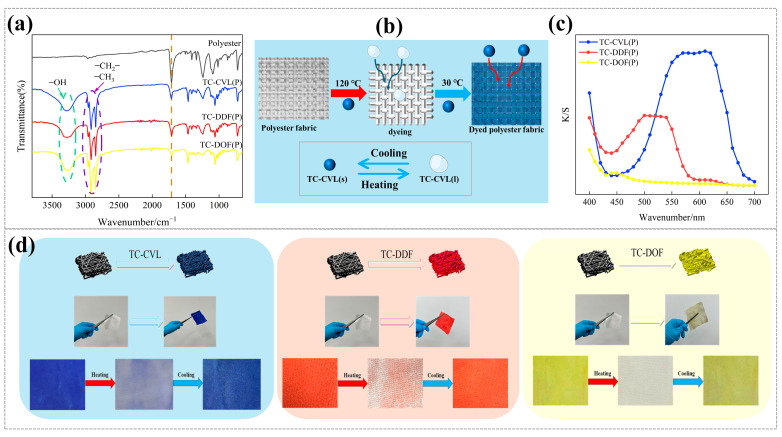
(**a**) FTIR spectra of both untreated and thermochromic polyester fabrics; (**b**) dyeing process methodology; (**c**) K/S values of three thermochromic polyester fabrics; (**d**) comparative analysis of fabrics pre- and post-dyeing, highlighting their reversible thermochromic properties under thermal cycling.

Tetradecanol was utilized as a solvent for multi-component thermochromic materials, offering excellent solid/liquid phase change properties along with being environmentally friendly, cost-effective, and readily available. Polyester fabrics were dyed by impregnation to produce three reversible thermochromic fabrics in blue, red, and yellow using TC-CVL, TC-DDF, and TC-DOF dyes, respectively (as shown in Figure 5b). Tetradecanol served as the dyeing medium, leveraging its solid/liquid phase change properties. 

FTIR spectroscopy analysis of the chemical structures of undyed polyester fabric and thermochromic polyester fabrics revealed that, in the spectra of thermochromic polyester fabrics (Figure 5a), the intramolecular and intermolecular hydrogen bonding of the three thermochromic dyes was observed at 3284 cm^−1^. Additionally, the vibration peaks of the endo-methylene and methylene groups of the thermochromic dyes were detected at 2912 cm^−1^ and 2848 cm^−1^. Compared to undyed polyester fabrics, the vibration peaks at 1712 cm^−1^ in thermochromic polyester fabrics were weakened, indicating that the stretching vibration of C=O in the lactone ring carbonyl of the three thermochromic dyes has a specific effect on the polyester fabrics. The variations in K/S values of the three dyed fabrics across different wavelengths are illustrated in Figure 5c. It was evident that the blue, red, and yellow fabrics exhibited their highest K/S values at their respective characteristic wavelengths. This indicated that each fabric achieved maximum color strength at specific wavelengths, corresponding to the peak absorption of the thermochromic dyes used. These findings highlighted the effectiveness of the dyeing process and the distinct optical properties of the thermochromic fabrics. 

At room temperature, the dyed polyester fabrics exhibited blue, red, and yellow, respectively. Upon heating, the polyester fabrics became colorless, demonstrating a significant thermochromic response. When the heating plate was turned off, the dyed polyester fabrics reverted to their original blue, red, and yellow with the temperature decreased, as illustrated in Figure 5d. After numerous cycles of heating and cooling, the dyed polyester fabrics consistently demonstrated the same reversible thermochromic effect as observed during the initial cycle. This stability in performance underscored the durability and reliability of the thermochromic dyes used. The fabrics maintained their ability to change color upon heating and revert to their original blue, red, and yellow upon cooling, indicating a robust and repeatable thermochromic response. These findings indicated the potential of these materials in applications requiring long-term reversible color changes, such as smart textiles and temperature-sensitive indicators.

### 2.3. Performance of Thermochromic Polyester Fabrics

**Figure 6:**
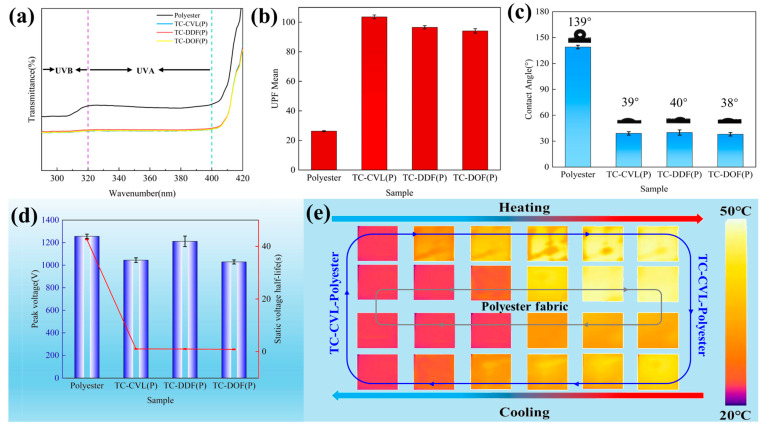
Performances of polyester fabrics before and after dyeing: (**a**) UV transmittance; (**b**) UPF values; (**c**) water contact angle; (**d**) peak voltage and static voltage half-life. (**e**) Infrared thermogram comparisons of polyester and thermochromic polyester fabrics (TC-CVL dyeing) under heating and cooling cycle.

Ultraviolet (UV) radiation is categorized into two types: UVB (ultraviolet B) and UVA (ultraviolet A). The UVA band, with a wavelength of 320–400 nm, UVB band, with wavelengths from 280 to 320 nm. UVB has distinct biological effects, particularly impacting the skin during outdoor activities. The anti-UV performance of dyed polyester fabrics was analyzed, as shown in Figure 6a,b. The results demonstrated a reduction in UV transmittance, indicating that these fabrics possess inherent UV resistance properties. By calculating the ultraviolet protection factor (UPF) values of the polyester fabrics before and after dyeing, it was found that the UPFs of polyester fabrics dyed by TC-CVL, TC-DDF, and TC-DOF were 103.61, 96.57, and 94.11, respectively (Figure 6b). In contrast, the UPF of the undyed polyester fabric was only 26.3. This significant increase in UPF values demonstrated the enhanced UV resistance of polyester fabrics due to the dyeing process with these thermochromic dyes. 

Polyester fibers are known for their poor hydrophilicity and inadequate hygroscopicity, which can lead to the accumulation of static electricity in dry environments. This static buildup results in the adsorption of dust and fluff, causing pilling and negatively affecting the fabric’s appearance. The application of multi-component biomass thermochromic dyes, utilizing tetradecane as a carrier, significantly enhanced the surface hydrophilicity of polyester fabrics. During the dyeing process, the hydroxyl groups of long-chain alcohols interact with the polyester fibers, improving their hydrophilicity. This interaction not only enhanced the moisture absorption capacity of the fabrics but also effectively reduced static electricity. Consequently, the dyed polyester fabrics exhibited better performance in terms of comfort and durability, making them more suitable for various applications where static resistance and moisture management are critical.

The contact angles of both undyed and dyed polyester fabrics were measured to assess their hydrophilicity. The water contact angle of undyed polyester fabrics was approximately 139°, indicating significant hydrophobicity. In contrast, the water contact angle of dyed polyester fabrics was reduced to 40° (Figure 6c), demonstrating that the application of reversible thermochromic dyes imparted considerable hydrophilicity to the polyester fabrics. This enhancement effectively addressed the inherent hydrophobic deficiencies of polyester fabrics. Furthermore, the antistatic properties of polyester fabrics before and after dyeing were evaluated (Figure 6d). Due to the high crystallinity, small amorphous zone, and low moisture return (0.4%) of polyester fabrics [27], there was a tendency for static charge accumulation on their insulating surfaces. The lack of polar hydrophilic groups also contributed to static electricity buildup [28,29,30]. Improved hydrophilicity reduced static charge accumulation, thereby enhancing the antistatic performance of the fibers. Static half-life measurements revealed that the static voltage of dyed polyester fabrics remained similar to that of undyed fabrics, around 1000–1200 V. However, the static half-life of dyed polyester fabrics was significantly reduced to less than 1 s, compared to 42.6 s for undyed fabrics. This substantial decrease indicated that the dyed polyester fabrics exhibited strong antistatic properties, making them more suitable for applications.

Based on the analysis of the above data, thermochromic dyes transitioned from solid particles to a liquid state and adhered to the fabrics as the temperature increased during the dyeing process. Upon completion of dyeing and subsequent washing, the dyes within the fiber voids and on the surfaces reverted to a solid state (Figure 5b). The inclusion of tetradecanol in the dye formulation imparted hydrophilic and antistatic properties to the fabric. The presence of dye particles within the fiber voids enhanced the UV resistance of the polyester fabrics.

Phase change materials (PCMs) have the ability to absorb or release substantial amounts of heat while maintaining a constant temperature during the phase transition. The infrared thermography of undyed polyester fabric and reversible thermochromic polyester fabrics is presented in Figure 6e. The initial temperature of the fabrics was set at 25 °C. Upon heating, the surface temperature of the undyed polyester fabric (inner ring in Figure 6e) increased significantly faster than that of the reversible thermochromic polyester fabric (TC-CVL polyester) (outer ring in Figure 6e), where the lighter colors indicated the higher temperatures. The reversible thermochromic polyester fabric demonstrated superior thermal regulation. During the cooling phase, the rate of surface temperature change in the TC-CVL polyester was lower, indicating its thermal insulation properties. This was primarily due to the phase change solvent in the thermochromic polyester fabric, which can store and release thermal energy through solid/liquid phase transitions, thereby endowing the thermochromic polyester fabric with effective thermal insulation capabilities [31]. 

**Figure 7:**
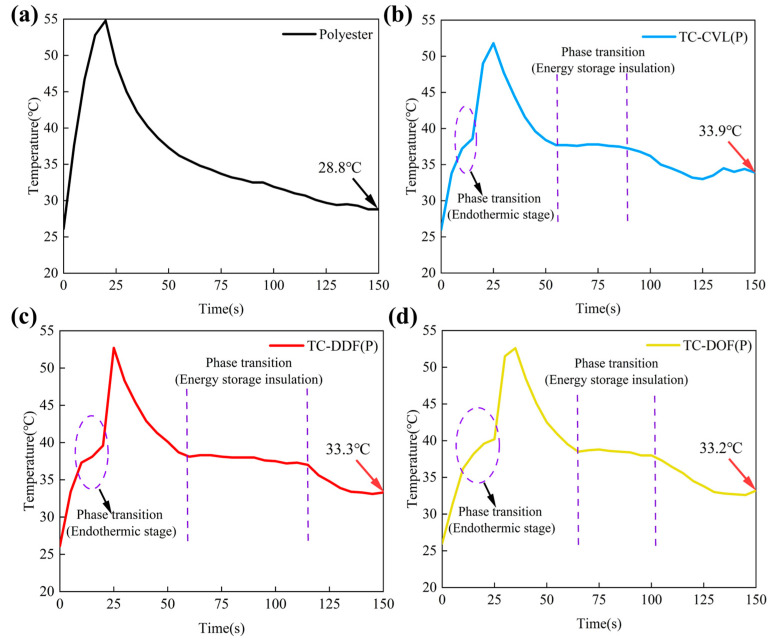
Temperature–time curve analysis for both polyester and thermochromic polyester fabrics: (**a**) polyester fabric; (**b**) TC-CVL-polyester fabric; (**c**) TC-DDF(P)-polyester fabric; (**d**) TC-DOF(P)-polyester fabric.

To further verify the thermal insulation and energy storage capabilities of thermochromic polyester fabrics, temperature–time curves were generated. Both undyed polyester and the three thermochromic polyester fabrics were placed on a heating plate, which was set at 55 °C. After heating, fabrics were permitted to cool for 150 s under ambient conditions (25 °C, 55% RH). An infrared thermal imager recorded the surface temperature of the fabrics in real-time, as shown in Figure 7. The results indicated that the temperature of the undyed polyester fabric increased linearly during the heating process (Figure 7a). In contrast, the polyester fabrics dyed with TC-CVL, TC-DDF, and TC-DOF thermochromic dyes exhibited an inflection point near 38 °C (Figure 7b–d). At this temperature, the rate of temperature change in the dyed fabrics decreased relative to the undyed fabric, primarily due to the solid/liquid phase transitions of tetradecane. During this phase transition, tetradecane absorbed a substantial amount of heat, stabilizing the temperature until the phase transition was complete. After this point, the temperature of the dyed fabrics continued to rise to approximately 55 °C.

Similarly, during the cooling process, the temperature of the fabrics dropped rapidly as the ambient temperature was lower than the fabric temperature. When the fabric temperature approached 38 °C, the cooling rate slowed significantly. This was because the ambient temperature (25 °C) was lower than the internal temperature of tetradecane, causing the thermal movement within the dye molecules to decrease. Consequently, tetradecane began to release heat and gradually solidified, resulting in a flattened cooling curve near 38 °C and a reduced cooling rate. The duration of this phase for the polyester fabrics dyed with TC-CVL, TC-DDF, and TC-DOF was 35 s, 57 s, and 38 s (between two dotted purple lines in Figure 7b–d), respectively, indicating that tetradecane was undergoing a liquid-solid phase transition during this period. Once the phase transition was completed, the temperature of the dyed fabrics continued to drop. After cooling for 150 s, the temperature of the undyed polyester fabric (28.8 °C) was lower than that of the dyed polyester fabric (33.9 °C, 33.3 °C, 33.2 °C) (Figure 7b–d). Under identical conditions, the dyed polyester fabrics exhibited the exothermic duration and the heat preservation effect at 38 °C. In contrast, the undyed polyester fabric demonstrated no energy storage or heat retention during the cooling process. Tetradecane, chosen as the phase change solvent for the three-component thermochromic dye, exhibited a solid/liquid phase change temperature of 37–38 °C, closely aligning with human body temperature. This characteristic made these thermochromic fabrics suitable for personal thermal management and intelligent thermal insulation applications.

## 3. Materials and Methods

### 3.1. Materials

Tea polyphenols were chemically pure and procured from Aladdin Chemical Reagents Ltd., (Shanghai, China). Crystalline violet lactone (CVL), 6′-(diethylamino)-1′,3′-dimethyl-fluoran (DDF), 3′,6′-dimethoxyfluoran (DOF), and tetradecanol (TD), all of chemically pure, were purchased from Sinopharm Chemical Reagent Co. Ltd (Shanghai, China). Polyester fabric was provided by Zhejiang Silk Technology Co. Ltd (Zhengjiang, China).

### 3.2. Preparation of Reversible Thermochromic Dyes

**Figure 8:**
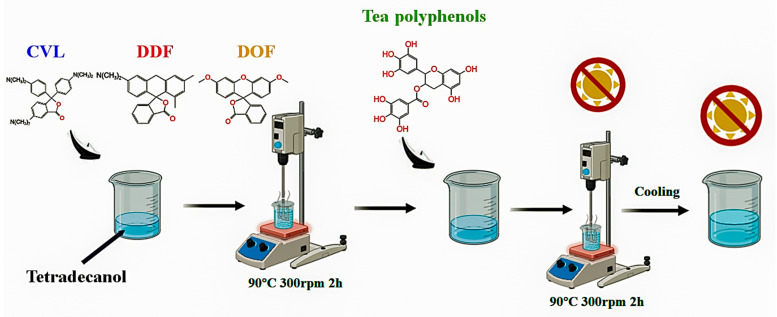
Schematic representation of the thermochromic dye synthesis process.

The materials listed in Table 1 were individually added to the dissolved state of tetradecane and subjected to stirring at 300 rpm for 2 h at 90 °C, ensuring the formation of a homogeneous solution. Subsequently, the tea polyphenol was uniformly incorporated into the above solution, followed by an additional 2 h of stirring at 90 °C. The mixture was then maintained at a constant temperature for 0.5 h before cooling to room temperature, resulting in the formation of biomass thermochromic dyes, as illustrated in Figure 8. These three biomass thermochromic dyes exhibited reversible thermochromic behavior, making them suitable for various applications in intelligent textiles. The synthesis process not only ensured the uniform distribution of the dye components but also enhanced the thermal stability and color-changing properties of the resulting materials.

### 3.3. Dyeing of Polyester Fabric Using Reversible Thermochromic Dyes

**Figure 9:**
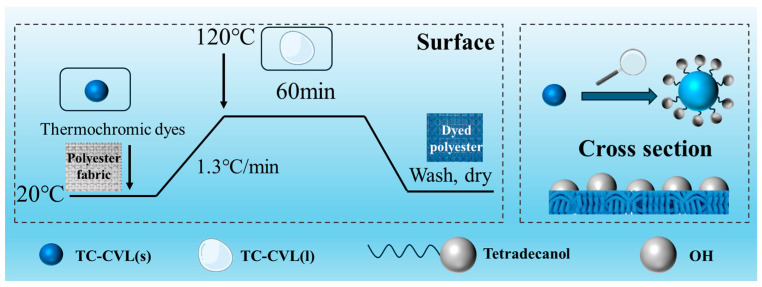
Dyeing process of thermochromic polyester fabrics.

The polyester fabric was dyed using the three prepared thermochromic dyes; the dyeing process is illustrated in Figure 9. After dyeing, the polyester samples were carefully extracted and subjected to a washing procedure for 10 min to remove any residual dye. Subsequently, the samples were air-dried at room temperature for 1 h. This methodical approach ensured the production of thermochromic polyester fabrics.

### 3.4. Characterization

To analyze the molecular structure changes of the three reversible thermochromic dyes before and after heating, several advanced spectroscopic techniques were employed. The absorbance of the biomass thermochromic dyes was measured using in-situ ultraviolet spectroscopy (PerkinElmer Lambda 950, Waltham, USA) at 25 °C and 50 °C, respectively, within the wavelength range of 400–700 nm. In situ Raman measurements (Ecopia Hall effect tester HMS-7000) were conducted at both 25 °C and 50 °C, covering the wavelength range of 300–3500 cm^−1^. Additionally, an in-situ Fourier transform infrared (FTIR) spectrometer (Thermo Nicolet iS50, Madison, WI, USA) was used to characterize three thermochromic dyes, obtaining spectra at 25 °C and 50 °C across the wavelength range of 400–4000 cm^−1^. High-resolution solid-state nuclear magnetic resonance (NMR) spectra were acquired using high-temperature solid-state NMR spectrometer (Agilent 600M, Santa Clara, CA, USA). A standard Agilent magic-angle spinning (MAS) probe with a 4 mm zirconium oxide rotor was employed for recordings at various temperatures on an Agilent DSX-300 spectrometer, facilitating the analysis of chemical structure changes and elucidating the color change mechanism of the biomass thermochromic dyes. The Datacolor SF650 Color Measurement and Matching instrument (10◦ standard viewer, CIE D65 Light Measurement, Datacolor, Lawrenceville, USA) was used to measure the K/S values of blue, red, and yellow fabrics at room temperature. The ultraviolet reflectance (UV-R) of the reversible thermochromic polyester fabrics was determined using a UV spectrophotometer with a resolution of 4 cm^−1^, in accordance with the European standard for sun-protective clothing (EN:13758-1 standard) [32]. The static water contact angle of the fabrics was measured using a Water Contact Angle Analyzer (Drop Shape Analyzer–DSA 25, Hamburg, Germany) with a 10 μL water droplet. The antistatic properties were evaluated using the YG—401 fabric inductive static tester according to the test methods for electrostatic propensity—Part 1: Test method using corona charging (GB/T12703.1–2021 standard) [33]. The evaluation involved measuring the peak voltage decay and decay time of fabrics through the corona charging method, with the acceleration voltage sat at 10 KV. An electric heating plate and an infrared thermal imager were employed to observe the color and temperature changes of the fabrics during heating and cooling cycles, thereby assessing their thermal responsiveness. 

## 4. Conclusions

In this study, three thermochromic dyes were prepared using biomass—derived tea polyphenols as color developers. The molecular structures of these dyes were analyzed before and after the thermochromic transition to elucidate the mechanism of color change. The results revealed that the bonds between the leuco dyes and the color developer dissociated upon heating and reformed upon cooling, thereby imparting reversible thermochromic properties. Additionally, polyester fabrics dyed with these reversible thermochromic dyes indicated that the water contact angle of the dyed polyester fabrics decreased from 139° to approximately 40°, demonstrating improved wettability. The UPF value of the dyed polyester fabrics increased from 29.3 to about 100, enhancing the UV resistance. Furthermore, a comparative analysis of the electrostatic voltage and electrostatic half-life of polyester fabrics before and after dyeing revealed a significant enhancement in antistatic properties. The electrostatic voltage half-life of undyed polyester fabric was measured at 42.6 s, whereas the dyed polyester fabrics exhibited a markedly reduced half-life of approximately 1 s. Additionally, infrared thermography demonstrated that the dyed polyester fabrics possessed notable energy storage and insulation capabilities. This method of fabricating thermochromic polyester textiles showed significant promise for the development of advanced smart color-changing textiles, especially for applications in personal thermal management and intelligent insulation. 

## Figures and Tables

**Table 1:** Components of three thermochromic dyes.

Samples	Leuco Dyes	Chromogenic Agents	Solvent
TC-CVL	Crystal violet lactone	Tea polyphenols	Tetradecanol
TC-DDF	6′-(Diethylamino)-1′,3′-dimethyl-fluoran	Tea polyphenols	Tetradecanol
TC-DOF	3′,6′-Dimethoxyfluoran	Tea polyphenols	Tetradecanol

## Data Availability

The experimental data used to support the findings of this study are included in the article.
